# Crystal structure of 2-{5-[2-(2-hy­droxy­phen­yl)diazen-1-yl]-1-methyl­pyrrol-2-yl}phenol methanol monosolvate

**DOI:** 10.1107/S2056989018007764

**Published:** 2018-05-31

**Authors:** Guiwen Yang, Huixiao Feng, Zhenming Yin

**Affiliations:** a College of Chemistry, Tianjin Key Laboratory of Structure and Performance for, Functional Molecule, Tianjin Normal University, Tianjin 300387, People’s Republic of China;, Key Laboratory of Inorganic-Organic Hybrid Functional Materials Chemistry, (Tianjin Normal University), Ministry of Education, Tianjin 300387, People’s Republic of China

**Keywords:** azo­pyrrole, crystal structure, hydrogen bonding

## Abstract

In the title azo­pyrrole compound, C_17_H_15_N_3_O_2_·CH_3_OH, the azo N=N bond adopts a *trans* configuration and the pyrrole N and azo group are in an *anti* orientation. The dihedral angles between the pyrrole ring and the two phenyl rings are 6.7 (3) and 54.7 (3)°. In the crystal, a supra­molecular ring structure is formed between two azo­pyrrole and two methanol solvent mol­ecules through four O—H⋯O hydrogen bonds.

## Abstract 2

The azo N=N bond adopts a *trans* conformation and the pyrrole N and azo group are in an *anti* orientation. The dihedral angles between the pyrrole ring and the two phenyl rings are 6.7 (3) and 54.7 (3)°. In the crystal, a supra­molecular ring structure is formed between two azo­pyrrole and two methanol solvent mol­ecules through four O—H⋯O hydrogen bonds.

## Chemical context   

Recently, azo­pyrrole dyes have received much attention for their promising use in the design of advanced materials and devices. For example, some thienyl­pyrrole azo dyes bearing heterocyclic groups have good non-linear optical properties (Raposo *et al.*, 2011 ▸). Mikroyannidis and coworkers found that many azo­pyrrole dyes are efficient bulk heterojunction solar cell materials (Sharma *et al.*, 2012 ▸). In a previous work, we reported the crystal engineering of some 5,5′-bis­(phenyl­diazo)­dipyrro­methane compounds and demonstrated their inter­locked type self-assemblies in the solid state *via* quadruple N—H⋯N hydrogen bonds (Yin *et al.*, 2008 ▸, 2009 ▸). In a continuation of this research, we report herein the crystal structure of 2-{5-[2-(2-hy­droxy­phen­yl)diazen-1-yl]-1-methyl­pyrrol-2-yl}phenol methanol monosolvate.
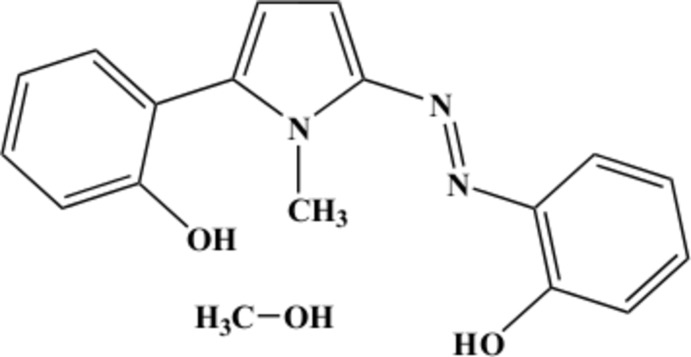


## Structural commentary   

**Figure 1:**
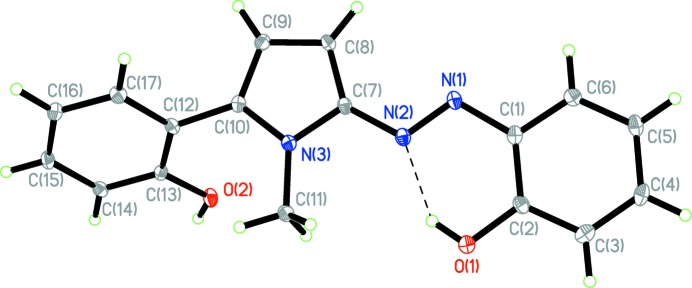
*ORTEP* diagram for the title compound, with displacement ellipsoids drawn at the 30% probability level. The methanol solvent mol­ecule was omitted for clarity.

The structure of the title compound is shown in Fig. 1 ▸. The asymmetric unit contains one azo­pyrrole mol­ecule and one methanol solvent mol­ecule. The azoylazo­pyrrole group is almost planar, reflected by the dihedral angle between the pyrrole ring (N3/C7–C10) and the benzene ring (C1–C6) of only 6.7 (3)°, which may be due to the existence of the intra­molecular O1—H1⋯N2 hydrogen bond (Table 1 ▸) between the hy­droxy group and the azo N atom. The dihedral angle between pyrrole ring and the other benzene ring (C12–C17) is 54.7 (3)°, which may be caused by the steric repulsion between hy­droxy group and methyl group. The azo N=N bond adopts a *trans* configuration and its length is 1.286 (2) Å, which is shorter than that in the crystal of 2,5-bis­(2-hy­droxy­phenyl­azo)-1*H*-pyrrole (1.293 Å; Li *et al.* 2009 ▸). It is worth mentioning that the N1 atom of the azo group and the N3 atom of the pyrrole ring are arranged on opposite sides with respect to the C7—N2 bond, which is the same as in the crystal of 2-phenyl­azo-1-vinyl pyrrole (Trofimov *et al.*, 2006 ▸) but different to many other observations (Li *et al.*, 2009 ▸; Yin *et al.*, 2008 ▸). The bond lengths in the pyrrole ring are more equal compared to those normally observed.

## Supra­molecular features   

**Figure 2:**
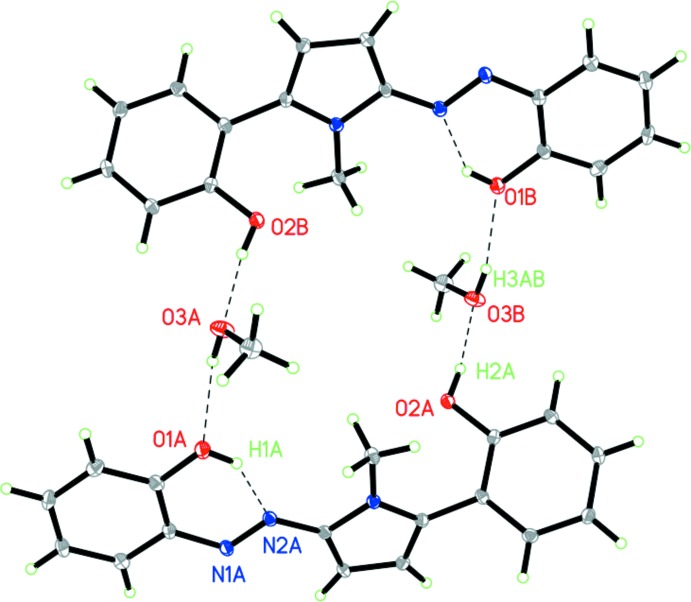
O—H⋯O hydrogen-bonded (Table 1 ▸) supra­molecular ring structure.

**Figure 3:**
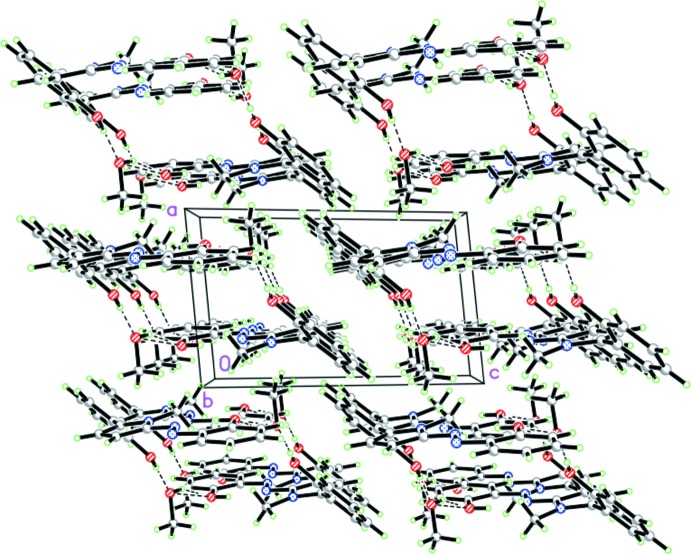
A view of the crystal packing along the *b* axis.

In the crystal, two azo­pyrrole mol­ecules are bridged by two methanol solvent mol­ecules through four O—H⋯O hydrogen bonds forming a large supra­molecular ring structure, in which the methanol acts as both a hydrogen-bond acceptor and a donor (Fig. 2 ▸, Table 1 ▸). This type of coordination environment is most populated (occupying 70%) for methanol mol­ecules as revealed by a search of the Cambridge Structural Database (CSD) (Brychczynska *et al.* 2008 ▸). The methyl groups point to the inside of the ring. The rings are further held together through C—H⋯π contacts involving the benzene rings (Table 1 ▸). There are no π–π inter­actions between the aromatic rings. The packing is shown in Fig. 3 ▸.

## Database survey   

A search in the Cambridge Structural Database (Version 5.38; Groom *et al.*, 2016 ▸) returned 45 entries for azo­pyrrole derivatives, including three entries for *N*-vinyl­phenyl­azo­pyrrole (Trofimov *et al.*, 2006 ▸; Rusakov *et al.*, 2007 ▸), four entries for mono- or bis­azo­pyrroles (Li *et al.*, 2009 ▸), two entries for azo calix[4]pyrroles (Nishiyabu *et al.*, 2006 ▸), five entries for pyrrole-azocrown ethers (Wagner-Wysiecka *et al.*, 2011 ▸; Szczygelska-Tao *et al.*, 2008 ▸), two entries for azo­pyrrole boron difluoride complexes (Li *et al.*, 2009 ▸; Lee *et al.*, 2012 ▸), ten entries for metal complexes (Li *et al.*, 2008 ▸; Li, & Dolphin, 2011 ▸; Yin *et al.*, 2012 ▸; Zhang *et al.*, 2015 ▸; Ghorui *et al.*, 2016 ▸); the majority are for mono- or bis­phenyl­azodipyrro­methanes (Yin *et al.*, 2008 ▸, 2009 ▸; Chen & Yin, 2014 ▸; Zhang & Yin, 2014 ▸).

## Synthesis and crystallization   

A 273 K solution of 2-amino­phenol 0.272 g (2.5 mmol) and aqueous HCl (2 mL) in water (2 mL) was treated with another 273 K solution of NaNO_2_ (0.18 g, 2.5 mmol) in 3 mL water, and the mixture was stirred at 273 K for 30 min. The diazo­nium salt solution was added dropwise to a solution of *N*-methyl­pyrrole (81 mg, 1 mmol) in aceto­nitrile (25 mL) and three drops of acetic acid. The combined solution was maintained at 273 K for 2 h with stirring. After that, EtOAc (25 mL) and water (25 mL) were added. The organic layer was separated and washed with water (20 mL) and dried with anhydrous MgSO_4_. The solution was evaporated and the residue was purified by column chromatography on silica (ethyl acetate/petroleum ether = 1:2), which gave the title compound as an orange powder (200 mg, 68%, m.p. 404 K).

^1^H NMR (400MHz, DMSO-*d*_6_): *δ* 3.73 (*s*, 3H, –CH_3_), 6.32 (*d*, *J* = 4 Hz, 1H, pyrrole C—H), 6.86 (*d*, *J* = 4 Hz, 1H, pyrrole C–H), 6.90–6.95 (*m*, 2H, Ar C-H), 6.99 (*t*, 2H, *J* = 8 Hz, Ar C—H), 7.23–7.31 (*m*, 3H, Ar C-H), 7.65 (*d*, *J* = 8Hz, 1H, Ar C—H), 9.95 (*s*, 1H, –OH), 10.43 (*s*, 1H, –OH) . Crystals suitable X-ray diffraction analysis were obtained by the slow evaporation of a CHCl_3_/CH_3_OH solution of the title compound.

## Refinement   

Crystal data, data collection and structure refinement details are summarized in Table 2 ▸. O—H atoms were located in a difference-Fourier map and refined freely. Other H atoms were positioned geometrically (C—H = 0.95 or 0.98 Å) and included in the final cycles of refinement using a riding model, with *U*_iso_(H) = 1.2*U*_eq_(C) or 1.5*U*_eq_(Cmeth­yl).

## Supplementary Material

Crystal structure: contains datablock(s) I. DOI: 10.1107/S2056989018007764/ff2153sup1.cif

Structure factors: contains datablock(s) I. DOI: 10.1107/S2056989018007764/ff2153Isup2.hkl

Supporting information file. DOI: 10.1107/S2056989018007764/ff2153Isup3.cml

CCDC reference: 1845021

Additional supporting information:  crystallographic information; 3D view; checkCIF report

## Figures and Tables

**Table 1:** Hydrogen-bond geometry (Å, °) *Cg*1 and *Cg*2 are the centroids of the C1–C6 and C12-C17 rings, respectively

*D*—H⋯*A*	*D*—H	H⋯*A*	*D*⋯*A*	*D*—H⋯*A*
O1—H1⋯N2	0.84	1.81	2.530 (2)	143
O2—H2⋯O3^i^	0.84	1.81	2.641 (2)	171
O3—H3*A*⋯O1^ii^	0.84	1.97	2.763 (2)	157
C11—H11*B*⋯*Cg*1^iii^	0.98	2.73	3.587 (2)	147
C18—H18*C*⋯*Cg*2^iv^	0.98	2.75	3.483 (3)	132

Symmetry codes: (i) 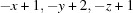; (ii) 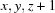; (iii) 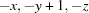; (iv) 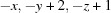.

**Table 2:** Experimental details

Crystal data	
Chemical formula	C_17_H_15_N_3_O_2_·CH_4_O
*M*_r_	325.36
Crystal system, space group	Triclinic, *P* 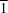
Temperature (K)	173
*a*, *b*, *c* (Å)	7.1597 (11), 9.8762 (16), 12.243 (2)
α, β, γ (°)	110.426 (3), 94.051 (3), 93.378 (3)
*V* (Å^3^)	806.0 (2)
*Z*	2
Radiation type	Mo *K*α
μ (mm^−1^)	0.09
Crystal size (mm)	0.14 × 0.13 × 0.12

Data collection	
Diffractometer	Bruker APEXII CCD
Absorption correction	Multi-scan (*SADABS*; Bruker, 2005 ▸)
*T*_min_, *T*_max_	0.987, 0.989
No. of measured, independent and observed [*I* > 2σ(*I*)] reflections	4168, 2830, 2071
*R*_int_	0.025
(sin θ/λ)_max_ (Å^−1^)	0.595

Refinement	
*R*[*F* ^2^ > 2σ(*F* ^2^)], *wR*(*F* ^2^), *S*	0.045, 0.109, 1.03
No. of reflections	2830
No. of parameters	222
H-atom treatment	H-atom parameters constrained
Δρ_max_, Δρ_min_ (e Å^−3^)	0.21, −0.23

Computer programs: *APEX2* and *SAINT* (Bruker, 2005 ▸), *SHELXS97*, *SHELXL97* and *SHELXTL* (Sheldrick, 2008 ▸).

## supplementary crystallographic information

### Crystal data

**Table:**

C_17_H_15_N_3_O_2_·CH_4_O	*Z* = 2
*M**_r_* = 325.36	*F*(000) = 344
Triclinic, *P*1	*D*_x_ = 1.341 Mg m^−^^3^
*a* = 7.1597 (11) Å	Mo *K*α radiation, λ = 0.71073 Å
*b* = 9.8762 (16) Å	Cell parameters from 1104 reflections
*c* = 12.243 (2) Å	θ = 2.3–28.1°
α = 110.426 (3)°	µ = 0.09 mm^−^^1^
β = 94.051 (3)°	*T* = 173 K
γ = 93.378 (3)°	BLOCK, yellow
*V* = 806.0 (2) Å^3^	0.14 × 0.13 × 0.12 mm

### Data collection

**Table:**

Bruker APEXII CCD diffractometer	2830 independent reflections
Radiation source: fine-focus sealed tube	2071 reflections with *I* > 2σ(*I*)
Graphite monochromator	*R*_int_ = 0.025
φ and ω scans	θ_max_ = 25.0°, θ_min_ = 1.8°
Absorption correction: multi-scan (SADABS; Bruker, 2005)	*h* = −8→8
*T*_min_ = 0.987, *T*_max_ = 0.989	*k* = −11→9
4168 measured reflections	*l* = −14→14

### Refinement

**Table:**

Refinement on *F*^2^	Primary atom site location: structure-invariant direct methods
Least-squares matrix: full	Secondary atom site location: difference Fourier map
*R*[*F*^2^ > 2σ(*F*^2^)] = 0.045	Hydrogen site location: inferred from neighbouring sites
*wR*(*F*^2^) = 0.109	H-atom parameters constrained
*S* = 1.03	*w* = 1/[σ^2^(*F*_o_^2^) + (0.0437*P*)^2^ + 0.2373*P*] where *P* = (*F*_o_^2^ + 2*F*_c_^2^)/3
2830 reflections	(Δ/σ)_max_ < 0.001
222 parameters	Δρ_max_ = 0.21 e Å^−^^3^
0 restraints	Δρ_min_ = −0.23 e Å^−^^3^

### Special details

**Table:**

Geometry. All esds (except the esd in the dihedral angle between two l.s. planes) are estimated using the full covariance matrix. The cell esds are taken into account individually in the estimation of esds in distances, angles and torsion angles; correlations between esds in cell parameters are only used when they are defined by crystal symmetry. An approximate (isotropic) treatment of cell esds is used for estimating esds involving l.s. planes.
Refinement. Refinement of F^2^ against ALL reflections. The weighted R-factor wR and goodness of fit S are based on F^2^, conventional R-factors R are based on F, with F set to zero for negative F^2^. The threshold expression of F^2^ > 2sigma(F^2^) is used only for calculating R-factors(gt) etc. and is not relevant to the choice of reflections for refinement. R-factors based on F^2^ are statistically about twice as large as those based on F, and R- factors based on ALL data will be even larger.

### Fractional atomic coordinates and isotropic or equivalent isotropic displacement parameters (Å^2^)

**Table:**

	*x*	*y*	*z*	*U*_iso_*/*U*_eq_	
O1	0.2218 (2)	0.53355 (15)	−0.07978 (12)	0.0254 (4)	
H1	0.2260	0.5941	−0.0113	0.038*	
O2	0.4830 (2)	1.13606 (14)	0.27561 (13)	0.0221 (4)	
H2	0.5527	1.1998	0.2636	0.033*	
O3	0.2725 (2)	0.68674 (17)	0.77244 (15)	0.0304 (4)	
H3A	0.2900	0.6375	0.8154	0.046*	
N1	0.2920 (2)	0.48800 (18)	0.14369 (15)	0.0191 (4)	
N2	0.2614 (2)	0.61395 (18)	0.14132 (14)	0.0181 (4)	
N3	0.2453 (2)	0.86125 (17)	0.24967 (14)	0.0166 (4)	
C1	0.2840 (3)	0.3796 (2)	0.03169 (18)	0.0180 (5)	
C2	0.2539 (3)	0.4013 (2)	−0.07572 (18)	0.0196 (5)	
C3	0.2592 (3)	0.2857 (2)	−0.18007 (19)	0.0247 (5)	
H3	0.2390	0.3001	−0.2526	0.030*	
C4	0.2933 (3)	0.1506 (2)	−0.17897 (19)	0.0243 (5)	
H4	0.2988	0.0728	−0.2508	0.029*	
C5	0.3199 (3)	0.1269 (2)	−0.07380 (19)	0.0235 (5)	
H5	0.3407	0.0329	−0.0735	0.028*	
C6	0.3159 (3)	0.2406 (2)	0.02965 (19)	0.0206 (5)	
H6	0.3353	0.2244	0.1015	0.025*	
C7	0.2752 (3)	0.7239 (2)	0.24820 (17)	0.0170 (5)	
C8	0.3197 (3)	0.7316 (2)	0.36227 (17)	0.0190 (5)	
H8	0.3478	0.6533	0.3870	0.023*	
C9	0.3152 (3)	0.8753 (2)	0.43346 (18)	0.0190 (5)	
H9	0.3386	0.9128	0.5164	0.023*	
C10	0.2708 (3)	0.9552 (2)	0.36319 (17)	0.0172 (5)	
C11	0.1888 (3)	0.8954 (2)	0.14600 (18)	0.0210 (5)	
H11A	0.1466	0.9933	0.1702	0.032*	
H11B	0.0858	0.8251	0.0986	0.032*	
H11C	0.2959	0.8908	0.0997	0.032*	
C12	0.2552 (3)	1.1127 (2)	0.39959 (17)	0.0167 (5)	
C13	0.3645 (3)	1.2011 (2)	0.35662 (17)	0.0166 (5)	
C14	0.3540 (3)	1.3501 (2)	0.39739 (17)	0.0184 (5)	
H14	0.4266	1.4092	0.3664	0.022*	
C15	0.2372 (3)	1.4124 (2)	0.48346 (18)	0.0203 (5)	
H15	0.2299	1.5144	0.5113	0.024*	
C16	0.1314 (3)	1.3269 (2)	0.52895 (18)	0.0211 (5)	
H16	0.0526	1.3699	0.5886	0.025*	
C17	0.1411 (3)	1.1790 (2)	0.48707 (18)	0.0201 (5)	
H17	0.0682	1.1207	0.5187	0.024*	
C18	0.0860 (3)	0.7274 (3)	0.7761 (2)	0.0310 (6)	
H18A	0.0712	0.7970	0.8541	0.046*	
H18B	−0.0020	0.6411	0.7594	0.046*	
H18C	0.0596	0.7720	0.7173	0.046*	

### Atomic displacement parameters (Å^2^)

**Table:**

	*U*^11^	*U*^22^	*U*^33^	*U*^12^	*U*^13^	*U*^23^
O1	0.0358 (9)	0.0192 (8)	0.0215 (8)	0.0050 (7)	0.0042 (7)	0.0068 (7)
O2	0.0216 (8)	0.0161 (8)	0.0286 (8)	0.0020 (6)	0.0093 (7)	0.0066 (7)
O3	0.0220 (8)	0.0362 (10)	0.0448 (10)	0.0055 (7)	0.0064 (7)	0.0279 (8)
N1	0.0188 (9)	0.0144 (9)	0.0233 (10)	0.0014 (7)	0.0030 (8)	0.0053 (8)
N2	0.0164 (9)	0.0155 (10)	0.0216 (9)	0.0004 (7)	0.0024 (7)	0.0057 (8)
N3	0.0157 (9)	0.0155 (9)	0.0188 (9)	0.0018 (7)	0.0022 (7)	0.0062 (7)
C1	0.0120 (10)	0.0167 (11)	0.0226 (11)	0.0005 (8)	0.0008 (9)	0.0040 (9)
C2	0.0165 (11)	0.0176 (12)	0.0253 (12)	0.0020 (9)	0.0015 (9)	0.0083 (9)
C3	0.0235 (12)	0.0296 (13)	0.0199 (11)	0.0021 (10)	0.0022 (10)	0.0072 (10)
C4	0.0209 (11)	0.0210 (12)	0.0236 (12)	0.0036 (9)	0.0026 (9)	−0.0015 (10)
C5	0.0212 (12)	0.0160 (12)	0.0304 (13)	0.0036 (9)	0.0001 (10)	0.0049 (10)
C6	0.0172 (11)	0.0211 (12)	0.0230 (11)	0.0008 (9)	−0.0021 (9)	0.0080 (9)
C7	0.0152 (10)	0.0136 (11)	0.0215 (11)	0.0004 (8)	0.0032 (9)	0.0052 (9)
C8	0.0222 (11)	0.0143 (11)	0.0216 (11)	0.0022 (9)	0.0025 (9)	0.0077 (9)
C9	0.0198 (11)	0.0184 (11)	0.0179 (11)	0.0012 (9)	0.0031 (9)	0.0051 (9)
C10	0.0143 (10)	0.0160 (11)	0.0195 (11)	0.0004 (8)	0.0028 (9)	0.0042 (9)
C11	0.0239 (12)	0.0176 (11)	0.0198 (11)	0.0019 (9)	−0.0034 (9)	0.0054 (9)
C12	0.0151 (10)	0.0142 (11)	0.0197 (11)	0.0000 (8)	−0.0022 (9)	0.0055 (9)
C13	0.0148 (10)	0.0174 (11)	0.0160 (10)	0.0031 (8)	−0.0001 (8)	0.0040 (9)
C14	0.0181 (11)	0.0154 (11)	0.0211 (11)	−0.0006 (8)	−0.0010 (9)	0.0066 (9)
C15	0.0210 (11)	0.0141 (11)	0.0227 (11)	0.0027 (9)	−0.0009 (9)	0.0031 (9)
C16	0.0206 (11)	0.0200 (12)	0.0216 (11)	0.0047 (9)	0.0048 (9)	0.0050 (9)
C17	0.0171 (11)	0.0202 (12)	0.0239 (11)	−0.0005 (9)	0.0028 (9)	0.0090 (9)
C18	0.0216 (12)	0.0363 (14)	0.0367 (14)	0.0030 (10)	0.0015 (11)	0.0153 (12)

### Geometric parameters (Å, º)

**Table:**

O1—C2	1.357 (2)	C7—C8	1.385 (3)
O1—H1	0.8400	C8—C9	1.386 (3)
O2—C13	1.362 (2)	C8—H8	0.9500
O2—H2	0.8400	C9—C10	1.389 (3)
O3—C18	1.416 (3)	C9—H9	0.9500
O3—H3A	0.8400	C10—C12	1.475 (3)
N1—N2	1.286 (2)	C11—H11A	0.9800
N1—C1	1.410 (2)	C11—H11B	0.9800
N2—C7	1.371 (2)	C11—H11C	0.9800
N3—C10	1.367 (2)	C12—C17	1.393 (3)
N3—C7	1.380 (3)	C12—C13	1.396 (3)
N3—C11	1.460 (3)	C13—C14	1.388 (3)
C1—C6	1.397 (3)	C14—C15	1.386 (3)
C1—C2	1.409 (3)	C14—H14	0.9500
C2—C3	1.390 (3)	C15—C16	1.382 (3)
C3—C4	1.375 (3)	C15—H15	0.9500
C3—H3	0.9500	C16—C17	1.378 (3)
C4—C5	1.389 (3)	C16—H16	0.9500
C4—H4	0.9500	C17—H17	0.9500
C5—C6	1.372 (3)	C18—H18A	0.9800
C5—H5	0.9500	C18—H18B	0.9800
C6—H6	0.9500	C18—H18C	0.9800
			
C2—O1—H1	109.5	C10—C9—H9	125.7
C13—O2—H2	109.5	N3—C10—C9	107.62 (17)
C18—O3—H3A	109.5	N3—C10—C12	124.35 (19)
N2—N1—C1	113.64 (18)	C9—C10—C12	128.03 (18)
N1—N2—C7	115.61 (18)	N3—C11—H11A	109.5
C10—N3—C7	108.49 (17)	N3—C11—H11B	109.5
C10—N3—C11	127.06 (17)	H11A—C11—H11B	109.5
C7—N3—C11	124.41 (17)	N3—C11—H11C	109.5
C6—C1—C2	118.50 (19)	H11A—C11—H11C	109.5
C6—C1—N1	115.80 (19)	H11B—C11—H11C	109.5
C2—C1—N1	125.66 (19)	C17—C12—C13	118.08 (19)
O1—C2—C3	119.0 (2)	C17—C12—C10	119.67 (19)
O1—C2—C1	121.41 (18)	C13—C12—C10	122.00 (19)
C3—C2—C1	119.6 (2)	O2—C13—C14	121.74 (19)
C4—C3—C2	120.4 (2)	O2—C13—C12	117.65 (18)
C4—C3—H3	119.8	C14—C13—C12	120.60 (19)
C2—C3—H3	119.8	C15—C14—C13	119.8 (2)
C3—C4—C5	120.6 (2)	C15—C14—H14	120.1
C3—C4—H4	119.7	C13—C14—H14	120.1
C5—C4—H4	119.7	C16—C15—C14	120.3 (2)
C6—C5—C4	119.3 (2)	C16—C15—H15	119.9
C6—C5—H5	120.3	C14—C15—H15	119.9
C4—C5—H5	120.3	C17—C16—C15	119.5 (2)
C5—C6—C1	121.5 (2)	C17—C16—H16	120.3
C5—C6—H6	119.3	C15—C16—H16	120.3
C1—C6—H6	119.3	C16—C17—C12	121.7 (2)
N2—C7—N3	117.55 (18)	C16—C17—H17	119.2
N2—C7—C8	133.93 (19)	C12—C17—H17	119.2
N3—C7—C8	108.49 (17)	O3—C18—H18A	109.5
C7—C8—C9	106.85 (19)	O3—C18—H18B	109.5
C7—C8—H8	126.6	H18A—C18—H18B	109.5
C9—C8—H8	126.6	O3—C18—H18C	109.5
C8—C9—C10	108.55 (18)	H18A—C18—H18C	109.5
C8—C9—H9	125.7	H18B—C18—H18C	109.5
			
C1—N1—N2—C7	−177.49 (17)	C7—C8—C9—C10	−0.8 (2)
N2—N1—C1—C6	−179.85 (17)	C7—N3—C10—C9	−0.6 (2)
N2—N1—C1—C2	2.6 (3)	C11—N3—C10—C9	177.13 (18)
C6—C1—C2—O1	179.88 (19)	C7—N3—C10—C12	178.51 (18)
N1—C1—C2—O1	−2.6 (3)	C11—N3—C10—C12	−3.7 (3)
C6—C1—C2—C3	−0.9 (3)	C8—C9—C10—N3	0.9 (2)
N1—C1—C2—C3	176.63 (19)	C8—C9—C10—C12	−178.2 (2)
O1—C2—C3—C4	179.32 (19)	N3—C10—C12—C17	128.7 (2)
C1—C2—C3—C4	0.1 (3)	C9—C10—C12—C17	−52.3 (3)
C2—C3—C4—C5	1.1 (3)	N3—C10—C12—C13	−57.2 (3)
C3—C4—C5—C6	−1.4 (3)	C9—C10—C12—C13	121.8 (2)
C4—C5—C6—C1	0.6 (3)	C17—C12—C13—O2	176.58 (17)
C2—C1—C6—C5	0.6 (3)	C10—C12—C13—O2	2.4 (3)
N1—C1—C6—C5	−177.19 (18)	C17—C12—C13—C14	−2.3 (3)
N1—N2—C7—N3	179.37 (17)	C10—C12—C13—C14	−176.47 (18)
N1—N2—C7—C8	1.5 (3)	O2—C13—C14—C15	−177.29 (17)
C10—N3—C7—N2	−178.23 (17)	C12—C13—C14—C15	1.5 (3)
C11—N3—C7—N2	3.9 (3)	C13—C14—C15—C16	0.0 (3)
C10—N3—C7—C8	0.2 (2)	C14—C15—C16—C17	−0.8 (3)
C11—N3—C7—C8	−177.69 (18)	C15—C16—C17—C12	0.0 (3)
N2—C7—C8—C9	178.4 (2)	C13—C12—C17—C16	1.5 (3)
N3—C7—C8—C9	0.4 (2)	C10—C12—C17—C16	175.89 (18)

### Hydrogen-bond geometry (Å, º)

Cg1 and Cg2 are the centroids of the C1–C6 and C12-C17 rings, respectively

**Table:**

*D*—H···*A*	*D*—H	H···*A*	*D*···*A*	*D*—H···*A*
O1—H1···N2	0.84	1.81	2.530 (2)	143
O2—H2···O3^i^	0.84	1.81	2.641 (2)	171
O3—H3*A*···O1^ii^	0.84	1.97	2.763 (2)	157
C11—H11*B*···*Cg*1^iii^	0.98	2.73	3.587 (2)	147
C18—H18*C*···*Cg*2^iv^	0.98	2.75	3.483 (3)	132

Symmetry codes: (i) −*x*+1, −*y*+2, −*z*+1; (ii) *x*, *y*, *z*+1; (iii) −*x*, −*y*+1, −*z*; (iv) −*x*, −*y*+2, −*z*+1.
